# Gut Microbiota and Obesity in Adults and Children: The State of the Art

**DOI:** 10.3389/fped.2021.657020

**Published:** 2021-03-19

**Authors:** Maddalena Petraroli, Eleonora Castellone, Viviana Patianna, Susanna Esposito

**Affiliations:** Paediatric Clinic, Department of Medicine and Surgery, University Hospital, University of Parma, Parma, Italy

**Keywords:** antibiotics, dysbiosis, gut microbiome, prebiotics, probiotics

## Abstract

In recent decades, obesity has become a serious public health problem affecting both children and adults. Considering the multifactorial origin of obesity, including modifiable factors, childhood was identified as the golden age for investing in obesity prevention by both promoting proper lifestyles and actively intervening in possible triggers. The gut microbiota is at the center of the most recent scientific studies and plays a key role in obesity development because it is intimately linked to energetic-humoral variations in the host: its alterations can promote a state of excessive energy storage, and it can be manipulated to maintain energy homoeostasis. This review aims to offer a panoramic understanding of the interplay between obesity and the gut microbiota, focusing on the contribution that the gut microbiota could have to the prevention of childhood obesity and its complications in adulthood. Currently, the use of some specific probiotic strains has been shown to be able to act on some secondary metabolic consequences of obesity (such as liver steatosis and insulin resistance) without any effect on weight loss. Although definitive conclusions cannot be drawn on the real impact of probiotics and prebiotics, there is no doubt that they represent an exciting new frontier in the treatment of obesity and associated metabolic dysfunctions. Targeted studies randomized on specific populations and homogeneous for ethnicity, sex, and age are urgently needed to reach definitive conclusions about the influence of microbiota on weight. In particular, we still need more studies in the pediatric population to better understand when the switch to an obese-like gut microbiota takes place and to better comprehend the right timing of each intervention, including the use of pre/probiotics, to improve it.

## Background

Once considered a problem specific to high-income countries, overweight, and obesity are now on the rise in low- and middle-income countries and becoming a large public health challenge. Obesity prevalence nearly tripled worldwide between 1975 and 2016. According to the most recent World Health Organization (WHO) report in 2016, more than 1.9 billion adults aged 18 years and older are overweight (39% of the World's adult population), and among them, over 650 million are obese (average 13%), with a major prevalence in the male population (1). Additionally, children and adolescents aged 5–19 years are involved in this growing global epidemic, with over 340 million overweight or obese individuals in 2016 (2).

The etiology of obesity has been attributed to several factors, including genetic susceptibility and environmental factors. Even considering all of them, the magnitude of this pandemic is still unexplained. Intervention on modifiable factors, such as lifestyle and diet, is the focus of national prevention policies: obese/overweight children are more prone to become adults affected by obesity and its consequences, such as cardiovascular diseases, type 2 diabetes, and cancer, so investing in children's health and healthy behaviors could be the key to tackling the problem (3, 4). A new point of interest is represented by the gut microbiota, which has been increasingly recognized as an important factor connecting genes, the environment, and the immune system (5).

The gut microbiota is involved in body weight control, energy homoeostasis, and inflammation, therefore playing a role in the pathophysiology of obesity (6). The human gut hosts a large number and variety of microorganisms. Among them, a population of ~1,000 species of bacteria is preponderant, with 99% of them belonging to ~40 species (7). All these microbes together form the so-called “gut microbiota,” to which we will simply refer with the term “microbiota.” In 2004, Backhet et al. first explored the ability of the gut microbiota to store energy from the diet and showed how, in developed and Western high-calories diet societies, this benefit became harmful (8). Subsequent studies were performed on *ob/ob* vs. germ-free and wild-type mice and obese vs. lean people to better understand whether there was some basic difference between the two populations. Controversies still exist about whether the difference between the gut microbiota of obese and lean people is more qualitative or quantitative and whether a subjective response to external stress is the origin or the consequence of altered energy metabolism. However, it has been established that while alteration of the gut microbiota can cause several health issues, including obesity, metabolic syndrome, diabetes, asthma, and atherosclerosis, gut microbiota manipulation could be a potential therapeutic target to reduce host energy storage (9–12). For these reasons, antibiotics and their abuse have been called into question, and considerable interest has focused on the use of probiotics associated with a balanced diet and adequate physical exercise (13, 14).

In this narrative review, we aim to fully explore the connection between the gut microbiota and obesity and to provide a panoramic understanding of the new frontiers of prevention of obesity through gut microbiota manipulation. PubMed was used to search for all of the studies published from the last 15 years using “Obesity” as a keyword associated with “Childhood,” “Gut Microbiota,” “Dysbiosis,” “Antibiotics,” and “Probiotics.” The search was limited to articles published in English that provided evidence-based data.

## The Influences of The Gut Microbiota On Energy Storage

After birth, the intestinal tracts of infants move gradually from poor to dense microbial colonization within the first 3 years of life and play a role in the maturation of the immune and endocrine systems. Mode of delivery, cessation of breastfeeding and introduction of solid foods are key factors driving the transition toward an adult-like gut microbiota (15). It is easy to understand why external factors at this time can influence an individual throughout their life.

Numerous animal models have consistently demonstrated that the gut microbiota can modulate host energy homoeostasis and adiposity through different mechanisms (Table 1), such as harvesting energy from the diet and modulating tissue fatty acid composition, secreting gut-derived peptides, and hormones with effects on the central nervous system (CNS), and inducing chronic low-grade inflammation by the release of lipopolysaccharide (LPS) (5, 12).

**Table 1:** Gut microbiota functioning and consequence of dysbiosis leading to obesity.

**Intestinal mechanisms**	**Normal function**		**Dysbiosis**	**Consequences**
Metabolic production	- Vitamin B12 and K synthesis - Promoter of biliar acids, entero-hepatic circulation - Complex carbohydrates fermentation in SCAFs: ↑ insulin sensitivity, ↓ deposition in adipose tissue, ↑ energy expenditure in other tissues	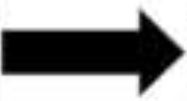	- ↓ production and/or absorption - Reducing bioavailability of substrates for VLDLs - ↑ and alterated SCAFs pattern - ↓ FIAF and AMPK	- Perniciousus anemia, emorragic risk - Reverse cholesterol transport - ↑ energy harvest - ↑ lipogenesis, fat storage, LPL and adipogenesis
Epithelium integrity and immunomodulation	- Influence on the cellular turnover - Modulation of gut biofilm - Modulator of gut epithelium permeability - Competition with pathogens for attachment sites and nutrients		- ↑ endocannabinoid system tone → ↑ gut permeability - ↓ defence against pathogens	- ↑ LSP absorption → endotoxemia - ↑ pro-inflammatory cytokines → ↑ lipogenesis, insuline resistance and impaired glucose tolerance
Hungry/Satiety	- ↑ production of GLP-1 and PYY by positive enteroendocrine L-cell - ↓ parasympathetic nervous system → ↑ leptine and ↓ ghrelin secretion		Alteration of satiety hormones equilibrium	- ↓ satiety, ↑ energy intake and harvest → ↑ gain weight

*SCAFs, short-chain acid-fatty; VLDL, very low density lipoprotein; FIAF, Fasting Induced Adipocyte Factor; LPL, lipoprotein lipase; LPS, lipopolysaccharide; GLP-1, glucagone-like-protein; PYY, Peptide YY*.

### Gut Microbiota, Adiposity, and Short-Chain Fatty Acids (SCFAs)

One of the basic tasks of the gut microbiota is the enzymatic conversion of primary bile acids into secondary bile acids, directly impacting the emulsification and absorption properties of bile acids (5, 12). Throughout this modification, gut microbiota influences biliary acid entero-hepatic circulation. Their secondary form binds G-proteins and stimulates the production of glucagon-like peptide 1 (GLP-1), reducing circulating, and hepatic levels of triglycerides. Qualitative and quantitative variations in the gut microbiota can alter this mechanism by promoting the accumulation of fats in the organism. For example, gut microbiota on a high-fat diet may convert dietary choline into hepatotoxic methyl-amines, reducing the bioavailability of choline, which is necessary for the assembly and secretion of very low density lipoproteins (VLDLs), and eventually promoting hepatic steatosis and lipoperoxidation (5, 12).

Moreover, “saccharolytic microorganisms,” the microbial community in the caecum and colon, are essential for processing dietary polysaccharides and fermenting them into SCFAs. There are three main SCFAs produced by different groups of bacteria: acetic acid (the most abundant in peripheral blood) is a substrate of cholesterol synthesis, an inducer of adipogenesis through the FFA2 receptor and a suppressor of appetite through central hypothalamic mechanisms; propionic acid is a precursor for protein synthesis, gluconeogenesis, and liponeogenesis in the liver, an inhibitor of fatty acid production and a low-grade inflammation-reducing agent in the gut; and butyric acid is a source for colonic epithelial cells and regulates gene expression through histone deacetylase inhibition (16).

SCFAs produced by fermentation may play a binary role as energy substrates and metabolic regulators. When they are bound to G-protein-coupled receptors (GPR41 and GPR43), they can modulate the regulation of gene expression at the transcriptional level in adipocytes, pancreatic β-cells, immune cells, and sympathetic neurons. Thus, SCFAs enhance insulin sensitivity and activate the sympathetic ganglion to prevent excess energy deposition in adipose tissue and enhance energy expenditure in other tissues, such as the liver and muscles (12). *GPR43*- and *GPR41*-deficient mice showed reduced GLP-1 secretion and impaired glucose *in vitro* and *in vivo*. Further, studies in *Gpr41*-deficient mice suggested that activation of GPR41 by SCFAs is responsible for the release of peptide YY (PYY), an anorexigenic peptide. In contrast, some studies on *Gpr43-*deficient mice have shown metabolic abnormalities, including excess fat accumulation, that are reversed when they are treated with antibiotics; just one study showed a lower body mass and a higher lean mass in *Gpr43-*deficient mice than in wild-type mice fed a high-carbohydrate/high-fat diet (16–19). The underlying reason for this inconsistency is still unclear. The microbiota may therefore influence the storage of peripheral adipose tissue and hence host adiposity: by the suppression of adenosine monophosphate kinase (AMPc) in the liver and muscles, the microbiota is able to influence fatty acid oxidation, ultimately leading to extra energy storage in these tissues. Moreover, inhibition of the fasting-induced adipocyte factor (Fiaf or angiopoietin-like protein 4, ANGPTL4) microbiota may lead to an increase in lipoprotein lipase (LPL) levels, promoting the storage of circulating triglycerides in adipocytes (5, 8, 12).

There is a general consensus from many studies, including research on humans, that the obese phenotype is associated with different profiles of SCFAs compared with the non-obese phenotype and that gut microbiota may directly and indirectly modulate processes related to obesity. However, there is an inside contradiction in the obese model: the abundance of substrates does not lead to an increase in the anorexic hormonal response, as expected. This dichotomy may indicate the involvement of additional particular bacteria or bacterial components/metabolites that may trigger regulatory cascades (12, 17, 18, 20).

### Gut Microbiota and the Control of Appetite

In addition to the regulation of digestion by different levels of the nervous system, there is ongoing communication from the gut to the brain in health and disease in the so-called “gut-brain axis” (21, 22). The gut microbiota not only communicates with nearby cells, but also generates and releases molecules that can signal to distant organs. In this sense, any modifications to it may also impact the regulation of the appetite. Regarding the central nervous system (CNS), gut bacteria and their metabolites may target it directly via vagal stimulation or indirectly through immune-neuroendocrine mechanisms (23, 24).

As mentioned before, SCFAs can modulate different levels, including gene expression and the release of hormones and peptides involved in appetite control. Glucagon-like peptide-1 (GLP-1) connects the nutritional load in the gut lumen to brain, liver, muscle, and adipose tissues by postprandial increases in satiety, gut transit time, and incretin-induced insulin secretion (12). The gut microbiota, by the production of non-digestible and fermentable fibers and SCFAs, demonstrated increased GLP-1 secretion in a mouse model and humans by influencing the expression of pro-glucagon, its precursor, and increasing the activity of GLP-1-positive enteroendocrine L-cells in the gut. Weight gain seems to decrease GLP-1 level (12, 24, 25).

PYY is also a hormone released by L-cells in the gut after feeding to reduce appetite and decrease gut motility. It works in synergy with leptin, while it has a complementary action to ghrelin. Gut microbiota, via SCFA-activated *GPR41*, induces stimulation of PYY, which in turn causes inhibition of gastric emptying, reduces intestinal transit time, promotes acetate and propionate absorption increasing energy harvest, and increases hepatic lipogenesis (24). However, the role of SCFAs in appetite regulation remains unclear. For example, different studies on acetate, the main SCFA secreted by intestinal bacteria and the preferred neuronal substrate, have shown its direct role in suppressing appetite via central hypothalamic mechanisms as well as its role in activating the parasympathetic nervous system accompanied by increased ghrelin secretion, hyperphagia, and obesity when it is produced by an altered gut microbiota (24).

Quality modifications in gut microbiota seem to significantly influence ghrelin and leptin levels. According to the mouse model, the abundance of several bacterial genera (*Mucispirillum, Lactococcus, Bifidobacterium*, and *Lactobacillus*, in particular) positively correlates with circulating leptin concentrations in mice, while other bacterial genera, including *Allobaculum, Clostridium, Bacteroides*, and *Prevotella*, negatively correlate with leptin levels. Regarding ghrelin, the predominance of the *Bifidobacterium, Lactobacillus*, and *B. coccoides*–*Eubacterium rectale* groups was negatively correlated; instead, *Bacteroides* and *Prevotella* positively influenced its levels (26).

### Gut Microbiota, Low-Grade Inflammation, and Intestinal Barrier Integrity

Over the past two decades, evidence of the link between obesity and inflammation has been established (27). Indeed, adipose tissue is not only a site of energy storage but also an active metabolic/endocrine organ involved in the secretion of adipokines, chemokines and pro-inflammatory cytokines such as tumor necrosis factor alpha (TNF-α), interleukin-6 (IL-6), plasminogen activator inhibitor 1 (PAI-1) and leptin, maintaining a state of chronic inflammation (12, 28). This activity may be enhanced by several external triggers, such as diet or the use/abuse of antibiotics, through alterations in the gut ecosystem.

Therefore, the gut microbiota plays a leading role, particularly due to the absorption of bacterial LPS and establishment of so-called endotoxaemia, as first demonstrated by Cani et al. in mice in 2007 (29).

LPS is an outer membrane component of gram-negative bacteria, which constitute ~70% of the gut microbiota (5, 6). When alterations in the gut microbiota occur, dysbiosis develops on behalf of gram-negative bacteria, and changes in gut barrier function may promote the release of bacterial endotoxins through damaged and leaky gut epithelium. In particular, the association of a high-fat diet with inflammation in obesity is correlated with metabolic endotoxaemia and a 2-3-fold increase in bacterial LPS levels in the blood (12, 29, 30). LPS is therefore significantly absorbed and, via TLR4/CD14, can activate various pro-inflammatory pathways and increase oxidative stress and insulin resistance in peripheral tissues such as the liver and muscles. Furthermore, the change in the composition of gut microbiota stimulates cannabinoid receptor type 1 (CB1 receptors) and consequentially the endocannabinoid system, which is the foremost responsible for the increased permeability of the intestinal barrier (5, 6, 12, 29, 30).

## Comparison of Leanness and Obesity

The human gut microbiota is composed of trillions of bacteria that specifically belong to *Firmicutes* (64% that includes *Bacilli, Clostridia* and *Mollicutes*) and *Bacteroidetes* (23% that comprise the genus *Bacteroides)* (31). Additionally, it is composed of *Proteobacteria* (8%), gram-negative bacteria (i.e., *Escherichia coli* and *Helicobacter pylori*), *Fusobacteria, Verrucomicrobia*, and *Actinobacteria* (3%) that include genera such as *Bifidobacterium* (31). The fact that the gut microbiota is involved in the development of obesity has been largely demonstrated by studies on an increase in total body fat in mouse germ-free models subjected to fecal transplantation from obese mice and adult humans. *Firmicutes* and *Bacteroidetes* are the two groups most heavily involved in microbial dysbiosis and the development of obesity: at the compositional level, obesity seems to be associated with changes in the *Firmicutes:Bacteroidetes* ratio (F/B), especially for a reduction in the relative abundance of *Bacteroidetes* that, in turn, contributes to excessive SCFA production and energy harvesting from colonic fermentation (8, 12, 32). The F/B ratio is often regarded as a marker of obesity in related studies. However, controversies still exist between studies conducted on human models, indicating that metabolic activity and not gut microbiota composition might be more relevant in obesity development (27). In 2014, Walters et al. concluded in a meta-analysis that there were no statistically significant differences in alpha diversity trends in microbiota composition according to z-score BMI or in the F/B ratio between obese and normal-weight adults across the multiple studies analyzed, even considering differences in sample handling and extraction or among the populations studied (33).

Recently, Abenavoli et al. critically analyzed several studies on the distribution and composition of the gut microbiota and its association with obesity in different subgroups, including children. They also concluded that even if the correlation between F/B and obesity constitutes a new milestone arising from three decades of research, current findings suggest deeper alterations in gut microbiota in obese patients than in that in lean subjects, requiring a better standardization of analytical methods (14). In children, the problem becomes even more complex because of the extraordinarily heterogeneous population by age and the intrinsically plastic gut microbiota, which could be the basis for therapeutic interventions in children to improve future overall health.

A great comparison of the gut microbiota of lean and obese children was carried out by Payne et al. (20). In contrast with adult studies, numerical variations in the bacterial population between obese and normal-weight children were not statistically significant for any population tested, and no correlation between the F/B ratio and childhood obesity was found. However, the analysis of fecal metabolite concentrations revealed a significantly lower concentration of intermediate metabolic products in obese children, suggesting utilization by obese gut microbiota. The final hypothesis proposed was that the increased F/B ratio observed in obese adults could be a result of dysbiosis findings in obese children arising from adaptation of individual microbial communities to long-term metabolic dysfunction (20).

Several studies confirmed the trace of increased substrate use activity, particularly in the detection of high concentrations of SCFAs in obese patients with varying profiles depending on the degree of dysbiosis. In particular, BMI *z*-score and the SCFAs acetate and propionate were significantly associated with gut microbiota composition at every taxonomic level. Their significantly higher concentration may indicate elevated colonic fermentation or, alternatively, decreased SCFA absorption due to low-grade inflammation or more rapid gut transit (16, 18, 23, 34).

*Staphylococcus* spp. and *Lactobacillus* spp. among the *Firmicutes* phyla and an *Actinobacteria* genus, *Bifidobacteria*, were studied in an attempt to find a predictive marker of childhood obesity (14). Indeed, during infancy, *Bifidobacteria* seem to remain higher in normal weight children, while *Staphylococcus aureus* stays at a lower level. Increased levels of *Lactobacillus* spp. and *Staphylococcus* spp., instead, seem to be associated with higher levels of plasma C-reactive protein contributing to low-grade inflammation, (35, 36). even if a positive correlation between high *Bifidobacteria* levels and elevated BMI was found in a study by Bai et al. (37) In children more than in adults, the link between the F/B ratio and obesity struggles to remain stable. Even if *Bacteroides* in the obese group was generally lower than that in the normal weight group, (34) the major and statistically significant difference between the obese and lean groups is the loss of abundance and diversity of gut microbiota in obese individuals (38, 39).

In 2018, European research studied the evolution of gut microbiota over 4 years in a group of 70 normal weight children at the starting point and the correlation with diet information, physical activity, and inflammatory markers (40). At the subsequent observations, 36 of the children gained excessive weight. By stool sample analysis, the authors established co-abundance associations of genera, and then they clustered the correlated bacterial taxa in four co-abundance groups (CAGs), superimposing them on the sample. Thus, they defined four clusters of children who had a significantly different gut microbiota layout from the others, with a gradual reduction in biodiversity and an increase in several inflammatory markers moving from the lean to the obese groups. More interestingly, when associated with 5 dietary groups, children who gained the most were those who followed higher carbohydrate diets, who had higher inflammatory markers and lower microbiota diversity (40). The exception was those who practiced high-intensity physical activity. The severity of the burden of childhood obesity is easily synthesized in the thriving of numerous studies conducted in unexplored and unexpected regions such as the Caribbean Islands (41), Northern Europe (42), South America (43), and East Asia (44) all questioning the link between gut microbiota and childhood obesity, searching for a marker of obesity and a way of prevention.

## Role of Diet and Antibiotics

When the abundance of keystone species in the gut starts declining, the symbiosis between host and gut microbiota collapses, a so-called dysbiosis is established and leads to a disruption of host metabolic health. Proposed mechanisms for dysbiosis include on one side a reduction in the number of bacteria that are metabolically protective against obesity and an increase in those able to extract more energy by indigestible polysaccharides (at the end, minor microbiota diversity); on the other, altered metabolic hunger/satiety pathway and reduction in intestinal barrier promoting low-grade-inflammation (45, 46).

Incorrect dietary habits are among the first triggers of dysbiosis. The large impact of diet is corroborated by studies carried out comparing breastfed children with those fed artificial milk. For example, the European IDEFIX study highlighted that exclusive breastfeeding until 6 months of age is protective against the development of obesity between 2 and 9 years of age (47). The protective effect seems to be linked to the composition of human milk, particularly the presence of bioactive substances such as leptin, insulin, GLP-1, PYY, and adiponectin, which are directly involved in the hunger/satiety balance (48). In addition, formula-fed infants are more prone to colonize with *Enterobacteriaceae* spp., *C. difficile, Bacteroides* spp., and *Streptococcus* spp. than breastfed infants, who are predominantly colonized by *Staphylococcus* spp., *Streptococcus* spp., *Lactobacillus* spp., and *Bifidobacterium* spp. Outside the lactation period, consumption of the typical “Western diet” (high-fat/high-sugar, low-fiber diet) has been shown to lead to dysbiosis, probably due to a reduction in diversity and, in particular, saccharolytic bacteria that produce SCFAs and microbes with anti-inflammatory properties (40, 49). Indeed, as clarified by Turnbaugh in 2008–2009 in animal models of the human intestinal ecosystem obtained by fecal transplantation, the switch from a low-fat/vegetable-polysaccharide-rich diet to a “Western” diet may modify the gut microbiota structure within a single day due to the increase in the relative abundance of *Firmicutes* at the expense of *Bacteroidetes* (resembling the changes seen in obese humans), altering not only the metabolic pathway but also the microbiome expression of genes involved in basic carbohydrate metabolism (50, 51).

Other external disruptors of host and gut microbiota symbiosis are represented by antibiotics. Antibiotics are the most prescribed and administered drug during infancy, due to the natural propensity of children to come into contact with new pathogens in order to develop their immune systems, some of them indirectly taken during intrauterine life (i.e., prophylaxis of vaginal group B *Streptococcus* and cesarean section delivery) or to prevent or treat bacterial infections typical of preterm infants (17, 52). However, they must be administered judiciously for two reasons that both have a tremendous impact on public health: first, to delay or better stop the widespread problem of antibiotic resistance; second, because of their side effects that occur by acting also on the symbiotic bacteria of the human organism, including the gut microbiota (53). In 2014, Azad et al. reported significantly greater odds of childhood overweight at age 9 and 12 years in boys exposed to antibiotics during the first year of life regardless of the drug dose and the number of courses, even after adjusting for relevant covariates (birth weight, family income, child, and maternal asthma) and measures of child diet and physical activity (54). Subsequently, two different cohort studies were performed with a large sample of children in relation to their prenatal exposure to antibiotics. In an American study, children exposed to antibiotics during the second or third trimester had an 84% higher risk of obesity, with a positive and significant association with BMI *z-*scores, waist circumference, and percentage of body fat (55). Furthermore, delivery by cesarean section, independent of prenatal antibiotic usage, was associated with a 46% higher risk of childhood obesity in offspring (55). Similarly, in a Danish analysis of school-age children, those who had been exposed to antibiotics in intrauterine life and in particular with a birth weight <3,500 g also became overweight (56).

Additionally, Saari et al., collecting data about weight, height, and drug purchase of 6,114 healthy boys and 5,948 healthy girls from birth to 24 months, concluded that antibiotic exposure before 6 months of age, or repeatedly during infancy, was associated with increased body mass in healthy children (57). In their study, the growth-promoting effect of antibiotics was also more pronounced in boys because of a significantly early exposure (57). Years later, Chinese multicentre cohort studies achieved similar results: repeated prenatal exposure to antibiotics was associated with childhood obesity at age 7 years, with a higher trend with an increasing number of antibiotic exposures (58). Recently, many systematic reviews and meta-analyses have been conducted on the link between antibiotics and childhood obesity by different international groups. The results suggest that antibiotic exposure during early life significantly increased the risk of childhood overweight and obesity (significantly increased the *z*-scores of childhood BMI and body weight), with consistent, though sometimes weak, associations with repeated treatments, early age of exposure (<6 months of age) and antibiotics with the broadest spectrum (13, 59–62). This trend lasts in later childhood according to some studies, particularly in relation to the number of antibiotic courses, regardless of the strength. In particular, in two different studies, the risk was higher in infants born to normal-weight mothers than in those born to obesity/overweight mothers, who had a decreased risk (59, 60).

Regarding various aspects concerning the gut microbiota, controversies also exist regarding the linkage between gut microbiota-antibiotics-obesity. For example, Gaber et al. in their original investigation of ~38,614 children (excluding those with complex chronic conditions or who received long-term antibiotics or multiple systemic corticosteroids), found no statistically significant difference in weight gain through age 7 years after exposure to antibiotics within the first 6 months of life compared with no exposure and discordant twins (61). In this study, the majority of exposed patients received antibiotics by 24 months of age and proportionally more broad-spectrum agents. Gaber et al. also analyzed previous studies, including those in developed nations, and proposed the existence of a “bias of generalization,” assuming standard results in populations with a high rate of child malnutrition (62).

## Probiotics and Prebiotics

The treatment of obesity cannot be separated from the maintenance of an adequate lifestyle. However, new discoveries about the gut microbiota are always arousing great fascination in the scientific community, which is wondering not only whether it can be used as an aid in the treatment of obesity but also whether it can prevent it. Indeed, if we could compensate from the outside the early alteration in gut microflora, we may even reverse the process. For that reason, prebiotics and probiotics and foods that contain them are seen as potential tools.

According to the WHO, a probiotic is a live microorganism which, when administered in adequate amounts, confers a health benefit on the host. It should be of human origin, safe, and free of vectors that are able to transfer resistance to antibiotics; it should have the ability to survive in the gut habitat, antagonize pathogens and stimulate the immune system (62, 63). A prebiotic is defined as a non-digestible fiber or non-digestible food ingredient that beneficially affects the host by selectively stimulating the growth and/or activity of one or a limited number of bacteria in the colon (64). It should be specified that at present, neither the European Food Safety Authority (EFSA) nor the US Food and Drug Administration has approved any probiotics or any product with health claims associated with probiotic administration for prevention and/or treatment of obesity because of insufficient characterization, undefined/non-beneficial claims, lack of relevant human studies, lack of measurable outcomes and finally, the quality of the presented studies (63). According to this policy, the most recent published review reports studies in animal models and adult humans in which prebiotics (inulin, oligofructose, and galactooligosaccharides) and probiotics (especially from *Bifidobacteria* and *Lactobacillus*, which have been given the status of Generally Recognized as Safe by the FDA) act as improvers of GPL-1 and PYY and as reducers of ghrelin secretion, and they can modify the sense of satiety, glucose sensitivity, and body composition through the re-establishment of regular microbiota activity (6, 65). Other studies do not fail to emphasize the constant conflict between some of them due to bias in the selection of the cohort, different doses, treatment duration and/or strains of bacteria (14, 66–68). As an example, Ejtahed et al. recently demonstrated that different strains of *Lactobacillus* had no significant effect on weight in either obese mice or rats. Actually, they reported trials in which probiotics were associated with weight gain, probably due to their ability to improve nutrient absorption and processing in the gut (69). Mazloom et al., questioning how effective probiotics were against obesity, found trials about the same strain of *Lactobacillus* with the opposite outcome depending on whether they were conducted on murine models or human subjects (70).

There are fewer studies in the pediatric population than in the adult population, and the peculiarities of single ages and, in particular, puberty play a great role in pediatric clinical trials. One example was the case with *Lactobacillus rhamnosus* GG. According to Luoto et al., the patterns leading to obesity are divided into two phases: an initial phase in the perinatal period until the age of 48 months and a second phase starting after 4 years of age. *L. rhamnosus* GG supplementation at 4 weeks before expected delivery and treatment of the child during the first 6 months of life determined a healthy growth pattern in children only until the end of the first phase, although no evidence of maintenance of the treatment effect was found at 10 years (71). In other trials, the use of prebiotics, probiotics or a combination of the two did not demonstrate an effect on weight loss but reduced comorbidities such as non-alcoholic fatty liver disease in children and adults (NAFLD). VLS3 (a combination of eight probiotic strains), consumed for 4 months by 48 children with non-alcoholic fatty liver disease, resulted in an improvement in liver lesions, likely because of a significant increase in GLP-1 concentration (72), while the administration of selected probiotics led to an improvement in liver function and metabolic parameters (73). Generally, probiotic supplementation could significantly improve liver steatosis, alanine aminotransferase, aspartate aminotransferase, total cholesterol and different inflammatory markers but could not ameliorate body mass index (74, 75).

Although definitive conclusions cannot be drawn on the real impact of probiotics and prebiotics, there is no doubt that they represent an exciting new frontier in the treatment of obesity and associated metabolic dysfunctions.

## Conclusions

The gut microbiota, the small intestinal microworld capable of influencing even human behavior (26, 76) has been identified as a new protagonist of energy homoeostasis, and its manipulations may be a possible key to solving the obesity problem. Our analysis of the literature shows that the differences recorded in the gut microbiota of obese individuals are secondary to incorrect and unhealthy lifestyles rather than to other idiopathic alterations.

Currently, the use of some specific probiotic strains has been shown to be able to act on some secondary metabolic consequences of obesity (such as liver steatosis and insulin resistance) without any effect on weight loss. The partial success of pre/probiotics in returning the gut microbiota to its original biodiversity may lie in an incorrect timing of action. The decisions made during pregnancy and the first years of childhood could consistently imprint the future functions of the gut microbiota and could be responsible for the conflicting responses to the treatments implemented. Early antibiotic exposure, mode of delivery and breastfeeding are the new frontiers of research, crossed with the use of pre/probiotics to stabilize beneficial effects and mitigate negative effects (77–81). Thus, there is the urgent need of targeted studies randomized on specific populations and homogeneous for ethnicity, sex, and age to reach definitive conclusions about the influence of gut microbiota on weight. In particular, we still need more studies in the pediatric population to better understand when the switch to an obese-like gut microbiota takes place and to better comprehend the right timing of each intervention, including the use of pre/probiotics, to improve it.

## Author Contributions

MP and EC co-wrote the first draft of the manuscript. VP performed the literature review. SE revised the manuscript and made substantial scientific contributions. All authors approved the final version of the manuscript.

## Conflict of Interest

The authors declare that the research was conducted in the absence of any commercial or financial relationships that could be construed as a potential conflict of interest.
